# First and second eye cataract surgery and driver self-regulation among older drivers with bilateral cataract: a prospective cohort study

**DOI:** 10.1186/s12877-018-0743-1

**Published:** 2018-02-17

**Authors:** Seraina Agramunt, Lynn B. Meuleners, Michelle L. Fraser, Kyle C. Chow, Jonathon Q. Ng, Vignesh Raja

**Affiliations:** 1 Curtin-Monash Accident Research Centre (C-MARC), Curtin University, GPO BOX U1987, PERTH, WA 6845 Australia; 2 Eye & Vision Epidemiology Research (EVER) Group, Perth, WA Australia; 3 School of Population and Global Health, The University of Western Australia, Perth, WA Australia; 4 Sir Charles Gairdner Hospital, Perth, WA Australia

**Keywords:** Older drivers, Bilateral cataract, Cataract surgery, Self-regulation, Contrast sensitivity

## Abstract

**Background:**

Driving a car is the most common form of transport among the older population. Common medical conditions such as cataract, increase with age and impact on the ability to drive. To compensate for visual decline, some cataract patients may self-regulate their driving while waiting for cataract surgery. However, little is known about the self-regulation practices of older drivers throughout the cataract surgery process. The aim of this study is to assess the impact of first and second eye cataract surgery on driver self-regulation practices, and to determine which objective measures of vision are associated with driver self-regulation.

**Methods:**

Fifty-five older drivers with bilateral cataract aged 55+ years were assessed using the self-reported Driving Habits Questionnaire, the Mini-Mental State Examination and three objective visual measures in the month before cataract surgery, at least one to three months after first eye cataract surgery and at least one month after second eye cataract surgery. Participants’ natural driving behaviour in four driving situations was also examined for one week using an in-vehicle monitoring device. Two separate Generalised Estimating Equation logistic models were undertaken to assess the impact of first and second eye cataract surgery on driver-self-regulation status and which changes in visual measures were associated with driver self-regulation status.

**Results:**

The odds of being a self-regulator in at least one driving situation significantly decreased by 70% after first eye cataract surgery (OR: 0.3, 95% CI: 0.1–0.7) and by 90% after second eye surgery (OR: 0.1, 95% CI: 0.1–0.4), compared to before first eye surgery. Improvement in contrast sensitivity after cataract surgery was significantly associated with decreased odds of self-regulation (OR: 0.02, 95% CI: 0.01–0.4).

**Conclusions:**

The findings provide a strong rationale for providing timely first and second eye cataract surgery for older drivers with bilateral cataract, in order to improve their mobility and independence.

## Background

Globally, driving a car is the most common form of transport among the older population in developed countries [1, 2] and plays an important role in their lifestyle [3]. Driving cessation has been associated with poorer physical, social and cognitive function as well as depression [4]. As adults are living longer and healthier lives [5] the number of older drivers on the roads will increase, which will have a significant impact on road safety [6].

Cataract is a common medical condition which increases with age and impacts on the ability to drive, increasing crash risk as well as driving difficulties [7–9]. To compensate for visual decline, previous research has found that some patients with cataract may self-regulate their driving while waiting for cataract surgery [10, 11]. Self-regulation refers to an older driver adjusting their driving in response to a perceived deterioration in their health, cognitive or functional abilities [12] which may result in a reduction in their driving or avoidance of specific driving situations [12, 13].

Surgery is a highly effective treatment for cataract. However bilateral cataract surgery is usually performed one eye at a time to avoid complications, such as endophthalmitis [14]. This means patients may be driving during the period between first and second eye surgery. To date, there is limited information about the impact of cataract surgery on driver self-regulation practices, specifically the separate effects of first and second eye surgery for bilateral cataract patients. While it is likely that first eye cataract surgery reduces the need for driver self-regulation it is unknown whether second eye surgery provides any additional benefits. As well, previous research has suggested that poor contrast sensitivity is strongly associated with driver self-regulation among the general older population [15–19]. It would therefore be useful to determine whether improvement in contrast sensitivity or other visual measures after first and second eye cataract surgery is associated with a reduction in driver self-regulation.

Naturalistic driving studies using in-vehicle monitoring devices can provide objective and accurate measures of driver self-regulation practices [20] and are able to capture participants’ real-life driving behaviour. A growing body of evidence comparing self-reported driving behaviour and naturalistic driving data has found that older drivers often misjudge their kilometres travelled, days driven per week as well as frequency of driving in challenging situations such as at night, in bad weather, in peak hour traffic and on highways [20, 21]. Therefore it is recommended that these devices be used to monitor driving outcomes rather than self-reported questionnaires alone [22]. To date, no study has used naturalistic driving data to examine changes in driver self-regulation behaviour throughout the cataract surgery process.

Therefore, the aim of this study is to assess the separate impact of first and second eye cataract surgery on driver self-regulation status, for bilateral cataract patients. A secondary aim of the study is to determine which changes in objective measures of vision are associated with changes in driver self-regulation status throughout the cataract surgery process.

## Methods

### Study design

A longitudinal prospective cohort study of older drivers with bilateral cataract was undertaken as part of the larger Cataract Extraction Driving Ability Research Study (CEDAR Study) [23]. A convenience sample of eligible participants were recruited consecutively from three public hospitals in Western Australia through two methods: direct invitation from ophthalmologists during their visit to the eye clinic or invitation letter from the researchers. From 290 eligible patients invited to take part in the study, 111 participated (38%) and 55 of these completed all three assessments.

### Eligibility criteria

The inclusion criteria were: a diagnosis of bilateral cataract; aged 55+ years; a current Western Australian driver’s licence; and driving at least twice a week. Exclusion criteria for participants were: a diagnosis of any significant eye conditions such as macular degeneration, glaucoma or diabetic retinopathy; a diagnosis of dementia, Alzheimer’s or Parkinson’s disease; wheelchair-bound; did not speak English or had previous cataract surgery.

### Data collection

Eligible participants were recruited between December 2014 and February 2017. Information was collected at three-time points for the participants: in the month before first eye surgery, at least one to three months after first eye surgery and at least one month after second eye surgery. Participants received a Participant Information Sheet and provided written informed consent before any data were collected, following the tenets of the Declaration of Helsinki. Ethics approval was obtained from the Curtin University Human Research Ethics Committee (Curtin University HR 29/2014), the Royal Perth Hospital Human Research Ethics Committee (Royal Perth #14–033), the South Metropolitan Health Service Human Research Ethics Committee (Fremantle Hospital #14–033), and the Sir Charles Gairdner Group Human Research Ethics Committee (Sir Charles Gairdner Hospital #2014–113).

### Questionnaires

Participants’ demographic characteristics were collected. As well, the Driving Habits Questionnaire (DHQ) [10] collected information on participants’ self-reported driving patterns, exposure and self-regulation practices in eight driving situations at the three assessments. The DHQ has been previously validated for use among a population of older drivers with bilateral cataract in Western Australia [24].

### Mini-mental state examination (MMSE)

General cognitive function was assessed using the Mini-Mental State Examination (MMSE) at the three assessments [25]. The inclusion criterion stipulated a score of at least 24 indicating normal cognitive function.

### Objective visual measures

Visual acuity, contrast sensitivity and stereopsis were assessed at the three time points by the researcher under the guidance of an ophthalmologist. A standardised protocol was followed under constant conditions and luminance. Participants wore their habitual corrective lenses or glasses used for driving for visual testing. Monocular and binocular visual acuity were measured at a distance of three metres using an Early Treatment Diabetic Retinopathy Study (ETDRS) acuity chart [26]. Letter by letter scoring was used and scores were converted to the logarithm of the minimum angle resolution (logMAR). Monocular and binocular contrast sensitivity were measured at 50 cm using the Mars Letter Contrast Sensitivity Test [27]. Scores were expressed as log units and participants were encouraged to guess the letters if hesitating, as directed by the protocol. Stereopsis was measured using the Titmus Fly Stereotest (Good-Lite Co., Inc.), measuring disparity from 4800 to 20 s of arc.

### In-vehicle monitoring device

A Geotab G06™ in-vehicle monitoring device with GPS log receiver was provided to the participants at the three assessments to record their naturalistic driving patterns for a period of seven days. The devices were connected either to the cigarette lighter for vehicles manufactured before 2006 or the On Board Diagnostic II (OBD II) port for vehicles manufactured after January 2006. The device can be easily inserted and removed and this was demonstrated to the participants. They were asked to disconnect the device if someone else drove the vehicle and move the device to any other vehicle they drove during the study period. Participants were also provided with a travel diary which was used to validate whether the participant was the driver of the vehicle for each trip. They were instructed to fill in the diary as soon as possible after the completion of each trip so their recall was accurate. Information collected included the type of vehicle driven, the number, age and position of passengers, purpose of the trip, date, start and finish time, odometer readings, trip duration and distance travelled. If they were unable to or forgot to disconnect the device when another person drove the vehicle, they were also asked to record this in the travel diary. After returning the device, each participant was interviewed to clarify any data issues, check their use of multiple vehicles and confirm whether there had been any other drivers of the vehicle while the device was connected.

The objective data obtained from the in-vehicle monitoring device included driving exposure, time and date of travel, speed, type of road and location. Night time driving was defined as the period between sunset and sunrise as obtained from the Australian Government Bureau of Meteorology website [28]; peak hour driving was from six to nine am and/or four to seven pm from Monday to Friday. Roads where there were more than 4000 vehicles per day per lane were defined as “heavy traffic roads” [29]. This information was obtained from Main Roads WA which is the State Government agency responsible for the road network in WA. To determine whether participants drove on highways/freeways, the researcher examined an interactive map provided by Geotab© which detailed each trip made by the participant.

### Classification criteria for driver self-regulation practices

Four driving situations were obtained from the self-reported DHQ which could be directly compared to the information obtained from the in-vehicle monitoring device. These four situations were used to classify participants as either self-regulating or non-self-regulating their driving in each situation. These situations included “*driving on highways/freeways”, “on heavy traffic roads”, “in peak hour traffic”* and *“night time driving*”. Initially, each of the four driving situations were examined separately to determine if participants’ self-regulated their driving in that situation. For example, participants were considered to have self-regulated their driving if they responded that they had not driven at night time based on information from the DHQ and the data from the in-vehicle monitoring device confirmed the same behaviour. Then all four driving situations were examined together and participants were classified as a “self-regulator” if they self-regulated their driving behaviour in at least one of the four driving situations. Otherwise, they were considered to be a “non self-regulator”.

### Statistical analysis

Descriptive statistics were used to describe the characteristics of the cohort. Repeated measures of analysis of variance (ANOVA) were used to assess the changes in the objective measures of vision. Cochran’s Q Tests were used to analyse the changes in driver self-regulation status in the four driving situations.

The outcome of interest was driver self-regulation status (self-regulator/non self-regulator). Two separate Generalised Estimating Equation (GEE) logistic models were undertaken. The GEE method is suitable for longitudinal or repeated measures study designs where observations within each participant are not independent [30]. GEEs permit specification of a certain working correlation matrix that accounts for this within-subject correlation, thus providing more robust regression coefficients. The first GEE logistic model analysed whether there was a significant change in self-regulation status after first and second eye cataract surgery, while controlling for potential confounding factors. The visual measures were not included in this model because vision changed as a result of the surgery.

The second model was undertaken to examine which changes in the three visual measures were associated with changes in driver self-regulation status. Potential confounding factors such as cognitive status (MMSE score), age group (55–64/ 65–74/ 75+ years), gender (female/male), marital status (single/married or de facto), retirement status (not retired/retired), and the number of comorbidities were entered in both models. All statistical analyses were performed using STATA 15®.

## Results

Fifty-five participants completed all three assessments resulting in 165 observations. Table 1 presents the baseline demographic characteristics of the cohort before first eye cataract surgery. Participants’ mean age was 73.3 years (SD =7.8) with 43.6% aged 75 years or older. The majority of participants were female (54.5%), married or in a de-facto relationship (61.8%), had completed a higher education degree (58.2%), lived with another person (54.5%) and were retired (76.4%). The mean score of 27.6 (SD = 2.2) on the MMSE indicated normal cognitive function. Participants also reported an average of 5.3 medical conditions (SD = 2.5) and an average of 50.9 years (SD = 9.5) driving experience at baseline.

**Table 1:** Baseline demographic characteristics of older drivers with bilateral cataract (*n* = 55)

Variable	n (%)
Age: mean (SD)	73.3 (7.8)
Age group (years)	
55–64	10 (18.2%)
65–74	21 (38.2%)
75+	24 (43.6%)
Country of birth	
Australia	21 (38.2%)
Not Australia	34 (61.8%)
Gender	
Female	30 (54.5%)
Male	25 (45.5%)
Marital status	
Single/separated/divorced/widowed	21 (38.2%)
De facto/married	34 (61.8%)
Retirement status	
Not retired	13 (23.6%)
Retired	42 (76.4%)
Living arrangements	
Alone	25 (45.5%)
Not alone	30 (54.5%)
Level of education	
Primary or Secondary School	23 (41.8%)
Higher Education	32 (58.2%)
Driving experience (years): mean (SD)	50.9 (9.5)
Number of comorbidities: mean (SD)	5.3 (2.5)
MMSE score: mean (SD)	27.6 (2.2)

*MMSE* Mini-Mental State Examination, *SD* standard deviation

Participants’ visual characteristics are presented in Table 2.

**Table 2:** Mean visual characteristics of older drivers before, after first and after second eye cataract surgery (*n* = 55)

Variable	Before surgery Mean (SD)	After first eye surgery Mean (SD)	After second eye surgery Mean (SD)	*P* value
Visual acuity (logMAR)^a^				
Better eye	0.18 (0.15)	0.10 (0.22)	0.00 (0.19)	< 0.001
Worse eye	0.39 (0.24)	0.36 (0.26)	0.11 (0.19)	< 0.001
Binocular	0.15 (0.15)	0.08 (0.21)	−0.02 (0.19)	< 0.001
Log contrast sensitivity (log units)^b^				
Better eye	1.57 (0.14)	1.62 (0.28)	1.68 (0.11)	< 0.001
Worse eye	1.41 (0.29)	1.47 (0.27)	1.61 (0.13)	< 0.001
Binocular	1.64 (0.14)	1.67 (0.25)	1.75 (0.08)	< 0.001
Stereopsis (log seconds of arc)^a^				
Binocular	2.14 (0.64)	2.31 (0.72)	1.96 (0.60)	0.002

^a^Lower scores represent better vision ^b^Higher scores represent better vision *log* logarithm*, logMAR* logarithm of the Minimal Angle of Resolution*, SD* standard deviation

Mean binocular visual acuity significantly improved from 0.15 logMAR (SD = 0.15) at baseline, to 0.08 logMAR (SD = 0.21) after first eye surgery and − 0.02 logMAR (SD = 0.19) after second eye surgery (*p* < 0.001).

Binocular contrast sensitivity significantly improved (*p* < 0.001) from 1.64 log units (SD = 0.14) before first eye cataract surgery, to 1.67 log units (SD = 0.25) after first eye cataract surgery and 1.75 log units (SD = 0.08) after second eye cataract surgery.

A significant change (*p* = 0.002) in stereopsis was found with stereopsis measuring 2.14 log seconds of arc (SD = 0.64) at baseline; worsening to 2.31 log seconds of arc (SD = 0.72) after first eye cataract surgery and improving to 1.96 log seconds of arc (SD = 0.60) after second eye surgery.

### Situations in which drivers self-regulated

Before first eye surgery, 47.3% of participants were classified as self-regulators in at least one driving situation. This reduced to 29.1% after first eye surgery and 18.2% after second eye surgery. In terms of the specific driving situations avoided, before first eye surgery, 12.5% of participants did not drive on heavy traffic roads, while only 8.3% and 2.1% did not drive in this situation after first and second eye cataract surgery respectively, representing a significant change (*p* = 0.020). Before first eye surgery, 37.0% of participants did not drive at night which decreased to 21.7% after first and 10.9% after second eye cataract surgery, which was significant (*p* = 0.002). There was no significant change in driver self-regulation status for driving during peak hour traffic (*p* = 0.100) and freeway/highway driving (*p* = 0.900).

### Multivariate analysis

The results of the logistic Generalised Estimating Equation (GEE) model examining changes in self-regulation status after first and second eye cataract surgery are presented in Table 3. The odds of being a self-regulator in at least one driving situation significantly decreased by 70% after first eye cataract surgery (OR: 0.3, 95% CI: 0.1–0.7) and by 90% after second eye surgery (OR: 0.1, 95% CI: 0.1–0.4), compared to before first eye cataract surgery, after adjusting for potential confounders. In addition, retired participants had 5.6 times the odds of self-regulating, compared to those who were employed (OR: 5.6, 95% CI: 1.1–27.7).

**Table 3:** GEE Logistic Model of the impact of first and second eye cataract surgery on self-regulation status

Variable	Odds Ratio	95% CI	*P* value
Cataract surgery			
Before first eye surgery	1.0		
After first eye surgery	0.3	0.1–0.7	0.004
After second eye surgery	0.1	0.1–0.4	< 0.001
Gender			
Female	1.0		
Male	0.4	0.1–1.3	0.122
Age group (years)			
55–64	1.0		
65–74	0.1	0.1–1.2	0.072
75+	0.7	0.6–8.0	0.737
Marital status			
Single	1.0		
Married/de facto	0.3	0.1–1.2	0.096
Retirement status			
Not retired	1.0		
Retired	5.6	1.1–27.7	0.036
Number of comorbidities	1.1	0.9–1.4	0.257
MMSE score	1.0	0.9–1.2	0.803

*CI* confidence interval*, GEE* Generalised Estimating Equation*, log logarithm, logMAR* logarithm of the Minimal Angle of Resolution*, MMSE* Mini-Mental State Examination*, SD* standard deviation

The results of the logistic Generalised Estimating Equation (GEE) model examining changes in the three objective measures of vision and driver self-regulation status are presented in Table 4. Improvement in contrast sensitivity after cataract surgery was significantly associated with decreased odds of self-regulating (OR: 0.02, 95% CI: 0.01–0.4). Males had significantly lower odds of being self-regulators (OR: 0.2: 95% CI: 0.04–1.0) and retired participants had significantly higher odds of being self-regulators (OR: 10.1, 95% CI: 1.8–54.8).

**Table 4:** GEE Logistic Model of change in visual measures and self-regulation status among older drivers with bilateral cataract

Variable	Odds Ratio	95% CI	*P* value
Gender			
Female	1.0		
Male	0.2	0.04–1.0	0.045
Age group (years)			
55–64	1.0		
65–74	0.1	0.1–1.4	0.091
75+	0.6	0.1–6.7	0.125
Marital status			
Single	1.0		
Married/de facto	0.6	0.2–2.6	0.523
Retirement status			
Not retired	1.0		
Retired	10.1	1.8–54.8	0.008
Number of comorbidities	1.2	0.9–1.5	0.174
MMSE score	1.0	0.8–1.3	0.882
Binocular visual acuity (logMAR)	2.5	0.2–26.9	0.455
Binocular contrast sensitivity (log units)	0.02	0.01–0.4	0.019
Stereopsis (log seconds of arc)	1.3	0.5–3.5	0.648

*CI* confidence interval*, GEE* Generalised Estimating Equation*, log* logarithm*, logMAR* logarithm of the Minimal Angle of Resolution*, MMSE* Mini-Mental State Examination*, SD* standard deviation

## Discussion

This is one of the first studies to use naturalistic driving information to assess the impact of first and second eye cataract surgery on driver self-regulation practices among a cohort of older drivers with bilateral cataract. We found a significant reduction in driver self-regulation in at least one situation after both first and second eye cataract surgery, compared to the month before first eye cataract surgery. The study also found that changes in contrast sensitivity were associated with the reduction in driver self-regulation after cataract surgery.

The results of our study are consistent with some of the limited existing research on the impact of cataract surgery on driver self-regulation. A population-based study from Sweden found that 40% of all drivers increased their driving frequency after first eye or bilateral cataract surgery [31]. In addition, this study and a more recent prospective study from Sweden reported that between 25% and 37% of all patients who ceased driving before first eye cataract surgery started to drive after first eye or bilateral surgery [31, 32]. However, an earlier study from the USA which followed cataract patients over a longer time period found that driving exposure (mileage) decreased over time in a similar fashion for those who had cataract surgery and those who did not [33]. It is therefore possible that decreased self-regulation observed in our study was perhaps a “rebound” effect with increased driving and less self-regulation occurring in the period immediately following surgery. Our study was unable to address the longer term impact of cataract surgery on driver self-regulation and this warrants further research.

Since previous studies combined participants who underwent only first, or both eye surgeries in the analyses, they were unable to measure the specific effects of first and second eye cataract surgery separately. Our study demonstrated that while first eye surgery had a large impact on reducing the need for driver self-regulation among bilateral cataract patients, second eye surgery also had a significant impact, reducing the odds of driver self-regulation by a further 20% compared to baseline. This suggests the importance of timely second eye cataract surgery for bilateral cataract patients.

Previous research also supports our findings on the association between contrast sensitivity and driving outcomes. Contrast sensitivity has been associated with changes in driving difficulty after first eye [24] and after second eye cataract surgery [34]. Among the general population, contrast sensitivity has also been associated with driver self-regulation and cessation [15, 16, 18, 19] as well as crash risk [35]. It should be noted that our cohort had better baseline vision, including contrast sensitivity than in previous studies examining the impact of cataract or cataract surgery on driving difficulty and self-regulation [10, 11, 36, 37]. Despite this, 47.3% of participants still felt the need to self-regulate their driving due to their vision while waiting for first eye surgery, representing a significant limitation for their mobility. In addition, the relatively small improvement in binocular contrast sensitivity from baseline to after second eye surgery was still associated with a significant reduction in driver self-regulation. Although driver self-regulation is necessary and positive for road safety, it nevertheless limits an older person’s mobility and independence in the community. It is well known that driving cessation can have a negative impact on their lifestyle [3], but evidence suggests that self-regulation without cessation may also increase depressive symptoms among the general older population [38] and cataract patients specifically [11]. Therefore, our findings suggest that first and second eye cataract surgery can have a significant positive impact on restoring the mobility of drivers with bilateral cataract, even if their visual impairment is relatively mild.

The study also found that both first and second eye cataract surgery significantly reduced driver self-regulation in two specific situations; driving at night and on heavy traffic roads. A previous study also found that night time driving was the most common situation avoided by older drivers awaiting first eye cataract surgery [11]. Previous research also reported that 36% of older drivers with cataract had difficulty driving on heavy traffic roads [10]. However, neither of these studies examined how self-regulation status changed throughout the cataract surgery process and relied on the DHQ questionnaire alone to assess driving difficulty, which might be subject to recall and social desirability bias.

Lastly, this study found that male cataract patients were less likely to self-regulate their driving than females and is consistent with previous research [39, 40]. Retired drivers were also more likely to self-regulate their driving possibly due to the fact they have more flexibility to choose when and where they drive than those who are employed [41].

The major strength of this study was the use of naturalistic objective driving data to examine self-regulation practices and associated changes in objective visual measures throughout the cataract surgery process. Naturalistic data provide valid information and are more accurate than self-reported questionnaires, which are prone to social desirability and recall biases [20]. However, there were several limitations. Participants’ naturalistic driving behaviour was only measured for a period of one week meaning this may not be representative of their overall driving patterns, although this time frame is consistent with some previous research [21, 42]. As well, the study was only able to measure four difficult driving situations and further research should include an extended range of driving situations which have been shown to be challenging among cataract patients such as driving in the rain and parallel parking [11].

## Conclusion

It is well known that driving provides older adults with mobility, independence and enhances quality of life [3]. The current study found that even among a cohort of cataract patients with better vision at baseline, a significant proportion self-regulated or restricted their driving while awaiting surgery. First eye cataract surgery significantly reduced driver self-regulation, with second eye surgery providing further reductions. This study provides a strong rationale for providing timely first and second eye cataract surgery for older drivers with bilateral cataract in order to improve their mobility and independence.

## Abbreviations

ANOVA: Analysis of variance
CI: Confidence interval
DHQ: Driving Habits Questionnaire
ETDRS: Early Treatment Diabetic Retinopathy Study
GEE: Generalised Estimating Equation
log: logarithm
logMAR: logarithm of the Minimal Angle of Resolution
MMSE: Mini-Mental State Examination
OBD II: On Board Diagnostic II
OR: Odds ratio
SD: Standard deviation
